# Qualitative research reporting in genetic counseling: A state‐of‐the‐art assessment and recommendations for enhancing methodological congruence and quality

**DOI:** 10.1002/jgc4.70160

**Published:** 2026-02-15

**Authors:** Tasha Wainstein

**Affiliations:** ^1^ Department of Medical Genetics University of British Columbia Vancouver British Columbia Canada; ^2^ British Columbia Children's Hospital Research Institute Vancouver British Columbia Canada

**Keywords:** knowingness, methodological congruence, qualitative research, quality, rigor, transparency

## Abstract

Qualitative research plays a growing role in advancing genetic counseling scholarship through deep insights into experiences, contexts, and systems that shape health outcomes. These perspectives can inform effective and equitable patient‐centered care. However, qualitative research's potential to inform practice and policy remains limited by persistent concerns with respect to quality. These limitations result from the application of quantitative ideals of quality that are being borrowed or forced onto non‐positivist research paradigms. Also, attempting to apply a single set of quality practices across a vast array of diverse qualitative paradigms is equally problematic. This review provides a comprehensive assessment of the reporting practices of qualitative research (including mixed methods research and open‐ended questions in quantitative surveys) published in the *Journal of Genetic Counseling* over a period of 1 year. Thirty‐four articles were evaluated using a subjective, values‐based framework consisting of methodological congruence, knowingness, and transparency. In addition to examples illustrating methodological (in)congruence, I also discuss a typology of qualitative research that lends itself to achieving methodological congruence, and reflective questions for researchers, reviewers, editors, and knowledge users aiming to facilitate improved quality and rigor in qualitative inquiry. The findings of this review suggest there is a need to shift how qualitative research is taught, conducted, reviewed, and published within the field of genetic counseling and other allied healthcare contexts. By fostering clarity without compromising creativity, we can elevate the credibility, utility, and impact of qualitative inquiry within genetic counseling scholarship and broader applied health contexts.

## 1 INTRODUCTION

Increasing acknowledgment of the value of qualitative forms of evidence has resulted in broader adoption of qualitative research approaches in healthcare research (Bradbury‐Jones et al., 2017; Braun & Clarke, 2023a, 2024a; Small, 2005; Tuval‐Mashiach, 2017; Vasileiou et al., 2018), including genetic counseling research (Wainstein et al., 2023). Despite these advancements, qualitative (genetic counseling) research continues to face challenges related to quality, which in turn perpetuates the questioning of its utility and limits its applicability in contributing to improving health and wellbeing at the practical and policy levels (Braun & Clarke, 2024b).

There are two related issues with respect to quality: the first is the continued assessment of the quality of qualitative research using quantitative standards (Morse et al., 2002). In healthcare research, there has been a long‐held commitment to the (post)positivist research paradigm (Vasileiou et al., 2018). Frequently, qualitative researchers will use quantitative and (post)positivist ideas about quality in the mistaken belief that it legitimizes their work for a non‐qualitative audience (Varpio et al., 2021). They may also feel pressured (either internally or through reviewers' and editors' feedback) to conform to the format of reporting quantitative work that is prevalent across healthcare journals, regardless of how this can impact qualitative ideals of quality (Clarke et al., 2025). Also, early career researchers and genetic counseling trainees are frequently only taught the application, assumptions, and language of quality from a quantitative methods perspective.

The second issue is that how quality is defined, operationalized, and assessed in qualitative research is a complex and nuanced problem. There have been many attempts to provide checklists, guidelines, standards, and frameworks as a means of evaluating qualitative reporting on its own merits rather than relying on quantitative ideas of quality (Braun & Clarke, 2024a, 2024b; Buus & Perron, 2020; Levitt et al., 2017; O'Brien et al., 2014; Tong et al., 2007; Tracy, 2010). However, qualitative inquiry is characterized by epistemological, ontological, axiological, and methodological pluralism (A glossary of terms is provided in Appendix S1). Providing a single, unified set of clear‐cut markers of rigor and quality^1^ for the conduct and review of such work is therefore extremely challenging (Tracy, 2010). Some may interpret this lack of standardization to imply that an “anything goes” approach is acceptable, when in fact it demands that we are heightened in our commitment to an overt portrayal of the quality of our work.

### 1.1 Purpose of the review

In a previous publication (Wainstein et al., 2023), I, along with my co‐authors, provided a robust theoretical basis for the use of qualitative methodologies in genetic counseling research. In the conclusion of that paper, we stated, “But despite this enthusiasm [for using qualitative methodologies], we believe that qualitative research methodologies as currently enacted in genetic counseling scholarship could stand to be given additional attention in the domains of education and training, as well as implementation” (Wainstein et al., 2023; p. 312). The intention of this review is therefore to bolster these efforts through a comprehensive assessment of the reporting of qualitative research being conducted in the field of genetic counseling. I hope to provide an evaluation of the quality of reporting of the research in the *Journal of Genetic Counseling* to sensitize researchers, reviewers, editors, and knowledge users to the need for considered and thoughtful qualitative research design as well as coherence and transparency of reporting, reviewing, and editing. I focus on the quality of reporting as it provides the only concrete lens through which to estimate the rigor of the research that has been conducted (Braun & Clarke, 2024b). This article is best suited for readers with some foundational knowledge in qualitative research within genetic counseling, as it assumes familiarity with key qualitative concepts and philosophies. Novice researchers may find it helpful to first read Wainstein et al., 2023 (or other introductory texts) for background context before engaging with this paper.

Ultimately, doing excellent research requires excellent methodology and this includes the reporting of that methodology in a coherent, transparent, and accurate way. Enhancing this aspect of genetic counseling research will have a ripple effect in enhancing the *Journal*, which in turn will contribute to “developing and consolidating the recognition of genetic counseling as a rigorous area of academic study” (Austin, 2024a; p. 258). As healthcare research continues to place value on knowledge synthesis as the highest form of evidence, efforts to improve the quality of qualitative research are essential in enhancing the chances of these works being included in systematic reviews and meta‐analyses. This inclusion in turn can heighten the likelihood of practical and clinical uptake of qualitative research, an important goal when we situate genetic counseling research within an applied health research context.

### 1.2 Author's positionality statement

It is important to acknowledge that this review has been conducted from a subjective standpoint. I am informed by my training in qualitative research and personal alignment with non‐positivist research values (i.e., researcher subjectivity is an asset; the role of participant voice should be central; knowledge is created through interpretation and collaboration). I also have professional experience with designing, conducting, reporting, reviewing, editing, and teaching qualitative research that I have brought to bear in this process. Further, my training and experience in qualitative research (and genetic counseling research) are embedded within Western ideas of knowledge and knowing and their attendant relational and ethical values.

Epistemologically and axiologically, I believe that the structure and nature of genetic counseling research questions are fundamentally action‐oriented and driven toward directing and improving clinical practice. However, there is broader alignment of the readership and contributors to the *Journal* with a wide variety of qualitative philosophical paradigms, reflecting the uniqueness of each research study as well as the researchers who conduct them (Austin, 2024b). This plurality informed my decision to evaluate publications across the qualitative spectrum (including the tangents of mixed methods research and open‐ended survey questions) in the hope of providing guidance more holistically.

Learning how to conduct qualitative research is complex and is frequently undertaken in a shortened timeframe concurrent to the project itself. This is further challenged by having to acclimate to a unique, frequently inaccessible language/jargon, which makes the possibility of problematic operationalization of these concepts likely (Braun & Clarke, 2024b). As a genetic counselor, I place value on my pedagogical skills and therefore hope to employ my training in communicating complex information in useful and meaningful ways.

## 2 REVIEW METHODOLOGY

This review constitutes a detailed evaluation of 34 papers (from a total of 103 original research articles across all types of inquiry) that were published in 2023 (volume 32, issues 1–6) in the *Journal of Genetic Counseling*. My choice to focus on this volume lay in wanting to provide a current perspective, though I recognize that qualitative methodologies and research more broadly are constantly evolving and require dedicated revisiting to remain up to date. My choice to focus on a single volume was pragmatic: this is a substantial enough collection to provide a comprehensive evaluation, while being contained enough to conduct the review in a timely fashion. However, it is important to acknowledge that examining a single volume of the *Journal* offers only a snapshot of reporting practices and underlying values, which may fall short of reflecting broader or evolving trends in the field. Publications which used qualitative methodologies in whole or in part (i.e., mixed methods studies and quantitative surveys using open‐ended questions) were chosen for evaluation. I first reviewed the titles and abstracts of each publication to ascertain the research design. If this information was not stated, I conducted a full‐text review to confirm. I included one publication in which I was a co‐author.

My process was informed by similar efforts in methodological review, particularly those conducted by Bradbury‐Jones et al. (2017) and Braun and Clarke (2023a, 2024c). My focus was to assess the quality of reporting, though I also briefly examined the ways in which qualitative research was used with respect to approaches, techniques, participant groups, and research areas of interest. This latter effort allowed me to contextualize the breadth of qualitative research being undertaken in genetic counseling research (“focused mapping” according to Bradbury‐Jones et al.) and structure my assessment of quality accordingly. Absent from this review is any quantification of quality as “poor,” “moderate,” or “good” that one might expect from a systematic review or meta‐analysis, or assessment of agreement with checklists or guidelines like the Consolidated Criteria for Reporting Qualitative Research (Tong et al., 2007) or the Reflexive Thematic Analysis Reporting Guidelines (Braun & Clarke, 2024a). After reading thoroughly and making familiarization notes on all included papers as a means of immersing myself in the data set, I conducted descriptive coding. This was done manually using the notes function in Adobe to identify the characteristics of each paper across the attributes of study design, data collection techniques, data analysis methods, participant group makeup, research question focus, and participant group sizes. I then made detailed notes specifically on the methodological (in)congruence of each paper before comparing these across papers. I used my familiarization notes and these more targeted memos to generate a rough outline of the patterns that I could discern across articles in terms of gaps in understanding with doing, reporting, reviewing, and editing qualitative work. Engaging in discussions with genetic counseling and research colleagues provided a valuable form of critical dialogue that enhanced my analysis of the data and strengthened my confidence in the interpretations I developed. Recursive attention to the literature allowed me to construct my notes into a comprehensive set of observations and strategies for enhancement in the form of this paper.

I have not cited papers included in the review to minimize the likelihood of identification (Braun & Clarke, 2024c). While my evaluation pertains directly to the authors' reporting of qualitative research in these publications, it is also pertinent to note that the reviewers and editors of these articles played an important role in constituting the final product. I therefore have attempted to highlight throughout this review aspects of importance for these latter audiences that could bolster quality further.

In assessing quality of reporting, I have relied on a subjective conceptualization of what constitutes good practice, in particular: methodological congruence, “knowingness,” and transparency. Methodological congruence^2^ refers to the conceptual alignment or fit of all parts (i.e., purpose, research questions, theoretical assumptions/paradigms, methods, and outputs) of the research inquiry (Levitt et al., 2017). Braun and Clarke conceptualize “knowingness” as, “… captur[ing] understanding of what you are doing, why you are doing it, and how you are doing it makes (conceptual) sense” (Braun & Clarke, 2024c). Finally, transparency^3^ refers to the researcher's responsibility to clear and open articulation of the research procedures and findings such that the knowledge user does not have to make assumptions about the rigor of the work (Tuval‐Mashiach, 2017). These three constructs are not mutually exclusive; for example, transparency requires that researchers first know what they are doing to relay this to the audience. They also have an inherent flexibility in that, for example, a researcher can be knowingly methodologically incongruent but still ensure quality of their reporting using transparency. I selected methodological congruence as the primary concept for evaluating quality at the outset of the review; it provided an innately practical framework to operationalize quality within this form of inquiry. Transparency and knowingness were added as additional concepts later in the process, based on the ongoing analysis as well as review of the literature, to refine how methodological congruence could be achieved.

## 3 CRITICAL REVIEW ANALYSIS

### 3.1 Characteristics of papers included in the review

Of the 34 papers reviewed, 21 had a purely qualitative research design, six were described as mixed methods research, and seven included open‐ended questions analyzed using qualitative methods in an otherwise quantitative survey.^4^ A full description of study characteristics can be found in Appendix S1 (Table S1). Some characteristics have totals greater than 34 since several studies used multiple data collection types/analysis methods and focused on more than one participant group.

Eighteen of the 34 papers exclusively used interviews as their data generation technique, though this number increased to 24 when accounting for studies that generated more than one type of data. Other less common data generation techniques included focus groups (*n* = 2), live chat transcripts (*n* = 1), and story completion prompts (*n* = 1).

The most frequent study participant group was genetic counselors (*n* = 16), with an additional five studies investigating genetic counseling students, and seven investigating other genetics or nongenetics healthcare professionals (including interpreters, volunteers, patient navigators, administrators, and hospital leadership). Patients or clients who sought genetic counseling services collectively accounted for a further 13 studies. These 13 studies represented seven patient participant groups (two each for cancer, prenatal, and carriers, and one for DTC‐GT); three parent participant groups; two minoritized participant groups (Disability community; LGBTQ+ community); and one sibling participant group. In 26 of the 34 studies, the first author was a genetic counseling trainee at the time of conducting the research. I assigned categories to capture the broad essence of research questions though I acknowledge the overlaps in these categories and that many papers may have addressed more than one. They are perspectives (*n* = 15), professional experiences (*n* = 7), lived experiences (*n* = 5), behaviors (*n* = 3), needs and concerns (*n* = 3), and opinions (*n* = 1). Perspectives papers dealt primarily with understanding individuals' or groups' interpretations of beliefs, ideas, or topics within an interpretive framework that included their values. “Professional” and “Lived” experience papers captured firsthand, embodied events, emotions, and reflections—those of genetic counselors in the context of their professional practice, and those of patients or individuals with genetic conditions recounting their personal experiences. Papers in the “behaviors” category explored actions taken in relation to, or in response to, specific activities or skills. “Opinion” papers reported on explicit expressions of judgment or preference regarding a topic.

These characteristics are notable for their lack of variety with respect to data collection techniques, participant groups, and focus areas. This trend is not necessarily unique to genetic counseling research (see, for example, the heavy reliance on one‐on‐one interviews across qualitative research: Nunkoosing (2005)). The preference for generating data through interviews among genetic counselors is not surprising given the substantial overlap in skillset with genetic counseling clinical practice (developing rapport, active listening, use of open‐ended questioning to elicit narratives, reflexivity about the role of the counselor/researcher in shaping the interaction). But, if we are to truly harness the benefits of qualitative research for advancing genetic counseling knowledge and praxis, we need to value the plethora of knowledge sources and ways of knowing that are made available to us using this form of inquiry (Thorne et al., 2016). Much of genetic counseling research is situated in real‐life contexts, highlighting that the realities we study are both tangible and shaped by social influences. As genetic counselors, we are trained to consider and synthesize multiple sources of evidence and knowledge (like health records, genetic test reports, family histories, and patients' narratives) in our clinical work. Our research would benefit from taking a similar approach to gain more fulsome understandings of these diverse realities.

### 3.2 A foundation for methodological congruence: Philosophical paradigms

The beliefs, assumptions, and principles that guide qualitative research (i.e., philosophical paradigms) were reported in only seven studies; in these rare instances, a justification or rationale for the appropriateness of the chosen paradigm was even rarer. In a few instances, vague terms such as “*explorative*” and “*descriptive*” were used in place of a substantive description of a paradigm. In a few others, phenomenology or grounded theory, typically considered to be methodologies, were framed as paradigms.^5^ Considerations of epistemology, ontology, and axiology allow researchers to understand social phenomena, choose research methods, analyze data, and determine the best ways to construct the outputs of the analysis (for a thorough review of these concepts, see Wainstein et al. (2023)). In addition, reporting a paradigm is fundamental to the assessment of methodological congruence since, “…qualitative research cannot be assessed on its own terms if the researcher has not been clear about what those terms are” (Braun & Clarke, 2024b). Failing to do so risks the likelihood of incorrect assumptions being made about the quality of the work and in particular the assessment of the work being based on conformity to quantitative values.

**FIGURE 1:**
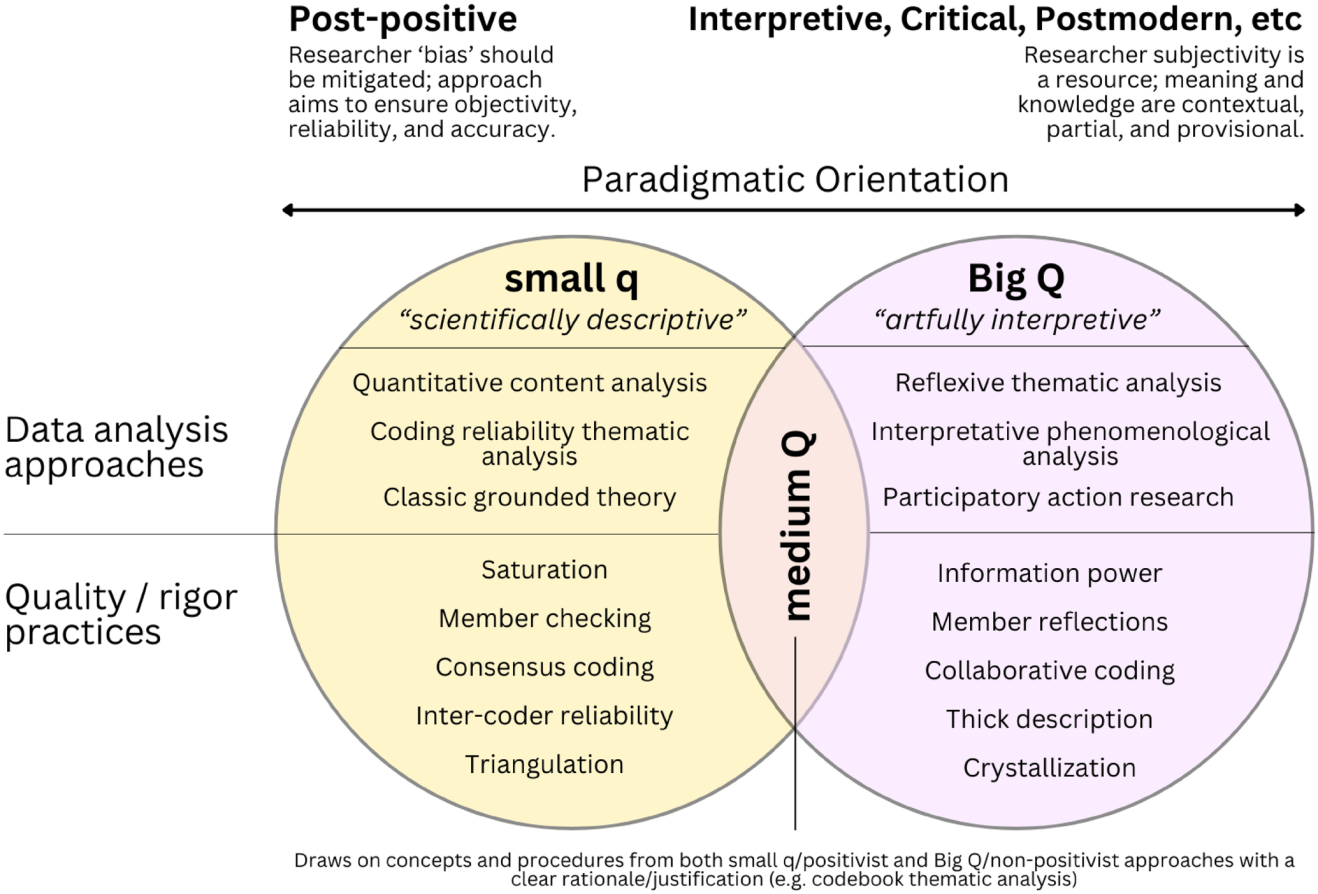
A typology of qualitative research as it relates to the concept of methodological congruence (adapted from concepts described by Braun and Clarke (2023b), Finlay (2021), and Kidder and Fine (1987)).

In centering methodological congruence as a marker of quality in qualitative genetic counseling research, it is helpful to consider a typology of qualitative traditions which has been described as a distinction between “small q qualitative and Big Q Qualitative” (Kidder & Fine, 1987). In small q qualitative research, the qualitative aspect is defined by collecting and analyzing data using qualitative techniques, but the underlying philosophical paradigms default to typically post‐positivist norms and are concerned with standardization and metrics that aim for objectivity and quantifiability (Braun & Clarke, 2023b, 2024b; Finlay, 2021; Kidder & Fine, 1987). Big Q Qualitative research involves both the use of qualitative techniques and the distinct values and traditions that have developed around qualitative methods (e.g., researcher subjectivity is a resource; knowledge is only ever partial and situated). A depiction of this typology is provided in Figure 1 as a means of demonstrating how methodological congruence can be achieved by first determining paradigmatic orientations and then enacting qualitative research studies in such a way that all aspects of the work align with either small q qualitative values or Big Q Qualitative values. Figure 1 also includes the possibility of an intermediate type, called “medium Q” which *knowingly and transparently* draws on concepts from both small q and Big Q approaches with a clear rationale or justification (Braun & Clarke, 2023b). Rather than adhering strictly to methodological rigidity purely for the sake of being theoretically grounded, medium Q approaches may constitute reasonable adaptations that allow us to answer questions confronting our profession in a pragmatic way that aligns with the goals of applied health research (Thorne et al., 2016). This approach should be considered distinct from seemingly unknowingly blurring together small q and Big Q features without careful consideration of necessity.

### 3.3 Participant group sizes, composition, justifications, and claims of generalizability

Participant group^6^ sizes across all studies ranged from 9 to 258 (Table S1). The size range for exclusively qualitative studies was 9–35; for the surveys was 16–165; and for the qualitative portions of the mixed methods studies was 14–146. These numbers are included here merely as a way of demonstrating the variability of study sizes being used in genetic counseling qualitative research. Assessing their appropriateness can only truly be achieved by evaluating them in the context of the epistemological, ontological, axiological, and methodological positions from which the research was conducted (further emphasizing the need to articulate paradigmatic orientations clearly). Nevertheless, it is possible to appreciate that those which were exclusively qualitative in their orientation had comparatively smaller size ranges, reflecting an overarching imperative to provide depth of analysis and rich, textured understandings (Sandelowski, 1995).

Generally, participant group composition and recruitment aligned with the goal of selecting the most knowledgeable informants to address the research questions posed and analytical goals (Sandelowski, 1995). But there were obvious caveats with respect to the diversity of the participant groups and the tendency to interrogate our own or our closest colleagues' perspectives (see Section 3.1).

The smaller participant group sizes that are normative in qualitative research do not release researchers from their obligation to provide justifications for their appropriateness as a means of enhancing rigor and quality (Boddy, 2016; Vasileiou et al., 2018). Nevertheless, justifications for participant group sizes and/or decisions about ending recruitment or data analysis were provided in only 13 of the 34 studies included in the review. The majority of studies that included a justification either named (data or theoretical) saturation^7^ or implied that this principle was invoked. Other justifications offered were theoretical sufficiency (Dey, 1999), information power (Malterud et al., 2016), or pragmatic considerations (Vasileiou et al., 2018). Some of the studies in which a justification was provided were unfortunately marred by either a lack of sufficient detail (e.g., “Interviews continued until data saturation was reached”) or convincing evidence supporting the claims (e.g., “Interviews were conducted until major recurring themes were emergent”).

Equally, there is a need to justify large participant group sizes. Failure to do so may surface assumptions about the ability to achieve an appropriate depth of analysis (Sandelowski, 1995), or may be construed as methodologically incongruent when they go against normative expectations of a particular method (e.g., interpretative phenomenological analysis) (Vasileiou et al., 2018).

Related to discussions of participant group size and sufficiency, is the concept of generalizability, or more precisely, the lack thereof in qualitative research. A lack of generalizability was cited as a limitation in 11 studies in this review (a further five studies indirectly referred to generalizability through the concepts of “representativeness” or “transferability”). Without further clarifying information, the term “generalizability” is typically understood to refer to *statistical‐probabilistic* generalizability which is a cornerstone of quality in quantitative research (Carminati, 2018; Hays & McKibben, 2021; Smith, 2018). Statistical‐probabilistic generalizability refers to the idea that a study's results can be extrapolated to a wider population because of the use of statistical sampling procedures. Qualitative inquiry that is ontologically and epistemologically aligned with the use of smaller participant groups to achieve depth and richness of subjective knowledge (e.g., constructivism) is therefore incongruent with this type of generalizability. Conversely, within a (post)positivist paradigm that does strive for breadth of inquiry, criticisms of sample size and generalizability may well be justified (Boddy, 2016). The incongruence between statistical‐probabilistic generalizability and Big Q research does not imply that the research findings from a non‐positivist study cannot be generalizable at the level of the phenomenon under investigation, or that non‐positivist researchers can absolve themselves of any responsibility to address the generalizability of their findings (Hays & McKibben, 2021). Rather, at the outset of their study, researchers could:

- First, consider whether generalizability is applicable for the analytic goals they hope to achieve (Sandelowski, 1995).
- If so, determine which type of generalizability (e.g., naturalistic, inferential, analytic, intersectional) is most appropriate (Hays & McKibben, 2021; Smith, 2018).
- In their reporting, explain how generalizability was operationalized in the research process, including their understanding of the role of knowledge users in determining generalizability (Carminati, 2018; Chenail, 2010; Smith, 2018).
- Use the report to help knowledge users connect meaningfully with the findings through thick description, connecting and contrasting findings with the literature, and including researcher reflexivity (overall and with respect to the implications or utility of their findings for practice) (Hays & McKibben, 2021).

### 3.4 Data analysis procedures

With respect to data analysis, the most common approach used was “thematic analysis” in nine studies, followed closely by “content analysis” in eight studies. Other less frequently reported data analysis methods included grounded theory (GT; *n* = 2), interpretive description (*n* = 3), and consensual qualitative research framework (*n* = 2). Of greatest concern were the 10 studies which did not name a specific data analysis approach and provided only a vague description (Table S1). Citations were frequently lacking for those which were more explicitly named. Describing and explaining the analytic approach with appropriate citation is a crucial component of qualitative research reporting, regardless of paradigmatic alignments. Although the intention here is not to aim for reproducibility as would be the case in a quantitative study, it is necessary to provide knowledge users with adequate information to assess methodological congruence.

It is also necessary to provide some form of justification or rationale for the choice of data analysis procedure and explain its appropriateness in answering the research question and achieving the goals of the inquiry. This type of justification was successfully accomplished in a minority of studies. For example, one study described using interpretive description (Thorne, 2013) because it aligned with their intentions to provide clinical/practical guidance on the topic. Another study used a reflexive thematic (Braun & Clarke, 2021a) approach to highlight the voices of a minoritized community (with representation from both the study participants and some of the members of the research team). For studies in which an established approach was used and cited appropriately, explanations and justifications for departures from or additions to typical procedures (e.g., using a codebook in reflexive thematic analysis) were infrequently provided.

In many instances, all variants of a group of data analysis approaches were treated as monolithic. This tendency was particularly evident in those studies which used “thematic analysis” (see Section 3.5), “content analysis,” and GT. Of the nine studies that used thematic analysis (TA), only one provided further description of the approach (e.g., coding reliability, codebook, or reflexive). Of the eight studies that used content analysis, two specified an inductive approach. Across both types of data analysis procedures, some studies provided descriptions that might allow knowing readers to infer these delineations in the absence of explicit statements. Each of these examples in fact represents an umbrella term for a group of approaches that have similarities in their procedures but vary with respect to philosophical underpinnings and goals or outputs of the inquiry. Types of GT, for example, include classic (positivist) GT (Glaser & Strauss, 2017), constructivist GT (Charmaz, 2014), situational analysis (Clarke, 2005), and critical realist GT (Lee, 2016). Aside from allowing for a more robust evaluation of methodological congruence, providing this level of detail within a description of one's approach to data analysis would also be helpful in demonstrating knowingness. A thorough review of GT in the context of genetic counseling research is described in Fishler Malone and Carmichael (2025).

### 3.5 A particularly problematic data analysis procedure: (reflexive) thematic analysis

“Thematic analysis” was the most frequently cited data analysis approach used in the reviewed studies and problematic in its enactment. Thematic analysis in fact refers to a family of approaches to data analysis which have some similarities but are also recognized as having essential differences in embedded philosophical assumptions, processes, and conceptualizations of themes (Braun & Clarke, 2021b). Therefore, describing a data analysis approach as “thematic analysis” without further qualification does not provide sufficient information to assess methodological congruence and is not transparent. The many publications which report the use of thematic analysis but their results appear to reflect something more akin to “categories” highlight the importance of thinking about how “themes” are conceptualized in these approaches. These distinctions are important among the different types of TA and in comparison to other pattern‐based approaches to analysis like content analysis and interpretative phenomenological analysis (Braun & Clarke, 2021b). In reflexive TA, themes are defined as capturing shared meaning underpinned by a central concept. In other types of TA, themes more closely resemble topic summaries or clusters of information. In inductive content analysis, the term “categories” is preferred and reflects broad areas or concepts that may be closely linked to interview questions (Vears et al., 2022). These differences in concepts are not trivial or merely semantic but can have consequences for how the outputs of qualitative research are valued (see Sections 3.9 and 3.10).

**FIGURE 2:**
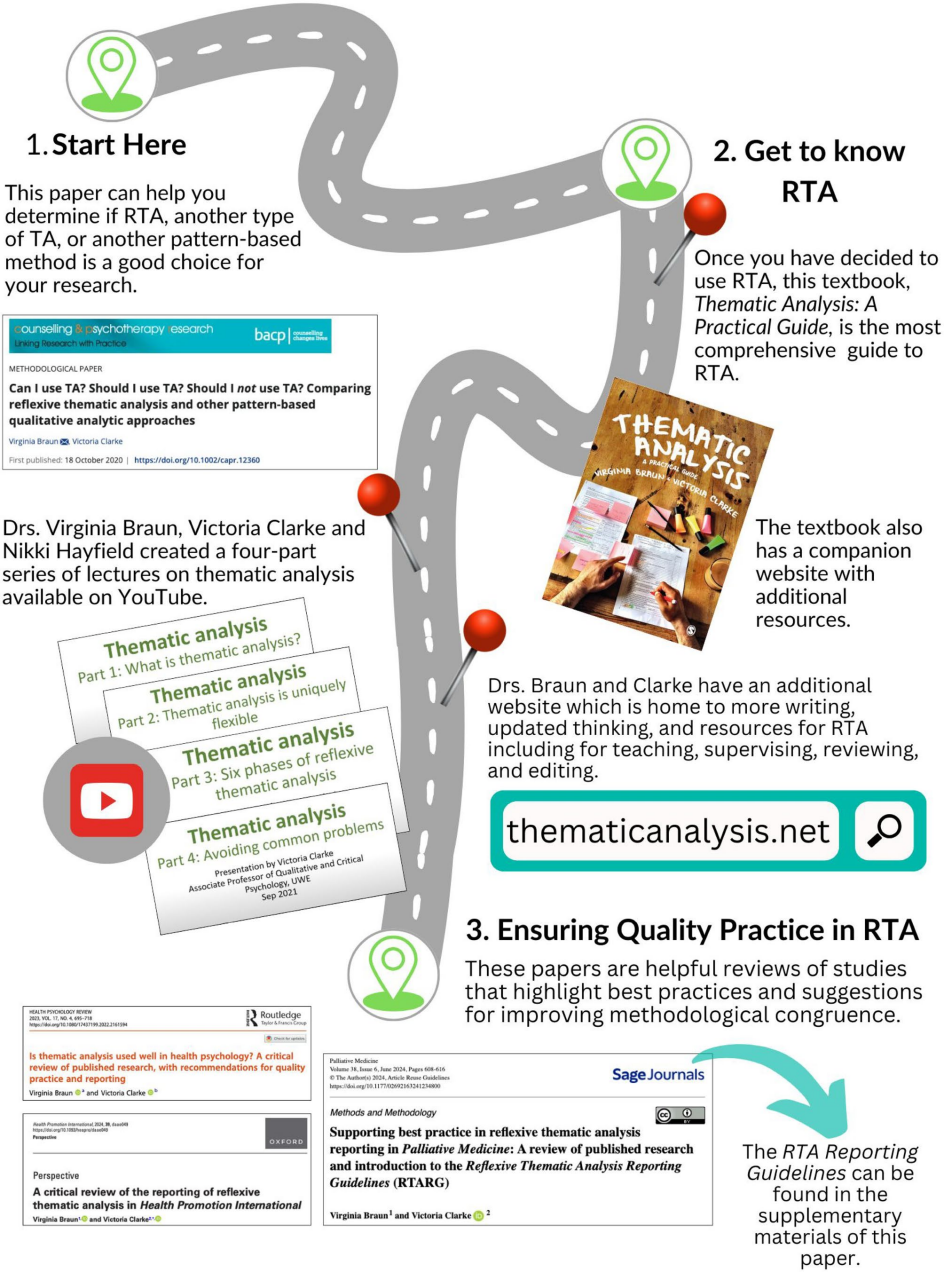
A roadmap for familiarizing oneself with the reflexive thematic analysis approach by Braun and Clarke (2021a, 2021b, 2023b, 2024a, 2024b, 2024c).

Reflexive thematic analysis (RTA) is one Big Q approach that has been thoroughly described and evaluated by its originators, Professors Virginia Braun and Victoria Clarke (Braun & Clarke, 2021a). Concerns relating to the use of RTA in their reviews of the approach are also evident in the genetic counseling studies reviewed here. In an attempt not to recapitulate their extensive body of work (Braun & Clarke, 2021c, 2021d, 2023a, 2024a, 2024b, 2024c), I have collated a roadmap shown in Figure 2 that intends to provide some orientation to thematic analysis generally, and the RTA approach specifically. Importantly, Braun and Clarke have recently published guidelines specific to enhancing the quality of reporting of RTA (Braun & Clarke, 2024a). Given the frequent use of thematic analysis and RTA, their wealth of resources should be considered essential reading for genetic counseling researchers, reviewers, editors, and knowledge users.

### 3.6 Quality and rigor practices

It is essential that all qualitative research is attentive to the need to demonstrate quality and rigor overtly. An abundance of strategies exists to achieve this goal, and many of these were reported in the examined literature, to varying degrees of success. The primary concern with the implementation of these strategies again lies within the disjuncture between the reported quality practices and the philosophical paradigms (either stated or assumed) within which the studies were framed. Table 1 provides a list of fictional studies that I have generated to illustrate common examples of methodological (in)congruence observed in this review with explanations of the specific contributors to (in)congruence.

**TABLE 1:** Fictional studies that demonstrate concepts related to, and common issues with, methodological (in)congruence.

Research question	Paradigm	Data gathering technique/s	Data analysis procedure/s	Quality/rigor practice/s or problem/s	Congruent?	Explanation
What are the long‐term psychological implications of the identification of Long QT syndrome as a secondary finding in adults?	Not stated	Digital diary entries	Thematic analysis	Use of a codebook, constant comparison, and bias bracketing	Indeterminate	Without the explicit statement of the paradigmatic orientation of the work or a specific type of thematic analysis, it is not possible to determine if there is methodological congruence. The stated quality practices also do not provide clarity, although bracketing might lead to the assumption of a positivist approach
What training do genetic counselors receive before starting a job in a laboratory setting?	Positivist	Open‐ended questions in a quantitative survey	Content analysis (Krippendorff, 2019)	Frequencies and measures of central tendency	Yes	The purpose of using this method is to enhance quantitative findings in a way that is concerned with objectivity, standardization, and quantifiability. The use of qualitative data collection techniques or analysis methods with alignment to positivist philosophies is therefore appropriate
How do parents make sense of a de novo pathogenic variant in the presence of a suggestive family history?	Constructivist	Semi‐structured, one‐on‐one interviews	Reflexive thematic analysis (Braun & Clarke, 2021a)	Consensus coding and intercoder reliability	No	Using multiple independent coders and measuring intercoder reliability is based on the belief that data have a fixed meaning that can be accurately captured. This contrasts with the view that data interpretation is subjective and influenced by researchers' perspectives, making the idea of a single ‘accurate’ coding method and the need for intercoder reliability irrelevant
What are parents' perspectives on the optimal post‐genetic/genomic testing supports for children with neuromuscular conditions?	Constructivist	Focus groups	Reflexive thematic analysis (Braun & Clarke, 2021a)	Collaborative coding for the purpose of educating a novice researcher	Yes	While coding in RTA can be sufficiently undertaken by a single researcher, the use of collaborative coding has been clearly justified and transparently reported in this case, therefore ensuring the methodological congruence of the work
What are the main concerns about fertility in young adults with cystic fibrosis?	Positivist	Unstructured, in‐depth interviews	Classic grounded theory (Glaser & Strauss, 2017)	Descriptive results formulated as themes without presentation of hypothetical probability statements	No	The use of classic grounded theory implies that a primary purpose of the study is to generate a theory. An analysis that is descriptive only, with no linking of concepts to form a theory fails to achieve methodological congruence (Vander Linden & Palmieri, 2021)
How is parental identity development affected by a positive NIPT finding?	Interpretivist	In‐depth interviews	Interpretative phenomenological analysis (Smith & Osborn, 2003)	Themes are descriptive, reflecting topics of conversation or broad strokes, rather than underlying meaning	No	An important quality indicator for IPA is that the study subscribes to the theoretical principles (i.e., that it is phenomenological, hermeneutic, and idiographic). The lack of an adequately evoked sense of experience and fine‐grained focus demonstrates that there is incongruence with the hermeneutic and idiographic nature of IPA (Nizza et al., 2021; Smith, 2011)
How do genetic counselors convey uncertainty in the context of a genetic test result?^a^	Pragmatic	Semi‐structured, one‐on‐one interviews	Interpretive description (Thorne, 2013)	Analysis moves beyond description and theorizing into interpretation; knowledge mobilization is built into the study's design logic	Yes	Interpretive description was designed for the purpose of conducting qualitative research in applied healthcare settings, the focus of which is to produce meaningful and actionable knowledge for the intended audience. An analysis that has disciplinary integrity and moves beyond basic description and theming to create an output that has utility in the real world of practice is the epitome of this approach
How do genetic counselors convey uncertainty in the context of a genetic test result?^a^	Constructivist	Recordings of genetic counseling sessions in which a variant of uncertain significance result is relayed	Theme‐oriented discourse analysis (Roberts & Sarangi, 2005)	Chronicling and narrating of the discourse analysis (DA) process and interpretation	Yes	One key marker of systematic and rigorous DA research is transparency, which in this case has been achieved through the careful documentation of the process of DA. This aligns with constructivism at the level of process, in addition to the epistemological positioning that discourse constructs, rather than reflects, reality (Greckhamer & Cilesiz, 2014)

^a^ These two rows intentionally address the same research question to demonstrate that methodological congruence can be achieved through multiple study designs.

A common manifestation of methodological incongruence, for example, was the use of (post)positivist reliability practices such as consensus coding, intercoder reliability, reporting of frequencies, triangulation, member checking, and saturation in constructivist studies (Braun & Clarke, 2021c, 2023a; Morgan, 2024; Smith & McGannon, 2018; Varpio et al., 2017). Conversely, there was infrequent use of first‐person writing style, inclusion of reflexivity and positionality statements, and recognition of the use of memos, field and analytical notes. These practices foreground the idea of researcher subjectivity and the role of the researcher in co‐creation of knowledge commonly associated with constructivist and other non‐positivist paradigms (Braun & Clarke, 2024b), though their use need not be limited to those paradigms (Wainstein et al., 2023; Zayhowski et al., 2025). In instances where positionality statements were included, there was a tendency to focus on researchers' roles in the study (e.g., as interviewers or coders) while not necessarily linking these with impact on study design, decision‐making, and analysis. Positionalities were also frequently framed in the context of steps taken toward mitigating implicit and explicit biases associated with the identities of the researchers, which would only be appropriate in (post)positivist settings. A recent publication addressing the use of reflexivity and positionality to enhance rigor in genetic counseling research (Zayhowski et al., 2025) can provide guidance on addressing these concerns.

### 3.7 Open‐ended questions in quantitative surveys

Open‐ended survey questions can be an important data gathering technique: they allow us to capture broader understanding of closed‐ended questions and can enhance anonymity so that sharing sensitive opinions feels more comfortable for respondents (Galura et al., 2022). The frequency of open‐ended questions in surveys may be explained by the popularity of Creswell's explanatory sequential design for mixed methods research (Creswell & Hirose, 2019; Creswell & Plano Clark, 2018), which is considered by some to be achievable through this data generation approach. Regardless of approach and despite their value, analysis of open‐ended questions has been reported to be lacking in rigor (Behr, 2015; Galura et al., 2022; LaDonna et al., 2018). The publications reviewed in this category represent a confused blend of content analysis and thematic analysis (e.g., data analysis approach was reported as “*thematic coding*,” or content analysis was named as the approach, but the results were presented as themes, not categories). It was exceedingly rare for any references to be cited for these methodological choices. Generally, these publications demonstrated alignment with small q approaches and incorporated some of the associated quality practices (e.g., consensus coding; intercoder reliability; reporting of frequencies).

Open‐ended survey questions are typically intended to be an adjunct to the primary quantitative survey, to explain or clarify responses to closed‐ended questions (Jackson & Trochim, 2002). Since answering open‐ended questions is a greater demand on survey respondents, answers tend to be abridged and incomplete (Scholz et al., 2022). There is no ability to probe responses further (as might occur in an interview or focus group format) and in some cases, responses are restricted in length through survey formatting (LaDonna et al., 2018). The depth and richness of these data are limited as a result, and the use of a small q/positivist framework (to quantify qualitative data) is therefore appropriate.^8^ Nevertheless, the responsibility to ensure quality and rigor remains; this includes making explicit the processes and procedures used and citing appropriately. Like all qualitative areas, there is no single approach for open‐ended questions, though quantitative content analysis is frequently employed (Lynch et al., 2023; Wainstein et al., 2023). For guidance about content analysis for open‐ended survey questions as well as its many other applications, a detailed review of (Coulston et al., 2025; Lynch et al., 2023; Vears et al., 2022) is recommended.

### 3.8 Qualitative considerations in mixed methods research

Mixed methods research describes an approach whereby both qualitative and quantitative methods are combined within a single study, including the collection, analysis, and integration of the two types of data (Creswell & Plano Clark, 2018). Although it was not stated in any of the articles reviewed, pragmatism is the paradigm most frequently associated with mixed methods research because of its emphasis on the practical integration of findings arising from both types of inquiry in a way that prioritizes an action‐oriented focus (Johnson & Onwuegbuzie, 2007).

In addition to this absence of clearly stated paradigmatic orientations, my findings closely mirror those I have described in the rest of this review for purely qualitative studies (i.e., insufficient citations for chosen data analysis approaches, justification for paradigmatic and methodological choices not provided, muddled use of both positivist and non‐positivist quality practices). Additionally, many of these papers did not specify the type of mixed methods design (i.e., convergent, explanatory sequential, or exploratory sequential), although in a minority of papers, it could be inferred from the description of the methods. This lack of specificity (or reliance on inference) further contributed to ambiguity and methodological incongruence. A common challenge among these papers was any attempt to integrate qualitative and quantitative findings meaningfully, despite integration being a defining feature of mixed methods research (Creswell & Plano Clark, 2018). Genetic counseling researchers contemplating undertaking *mixed methods* studies should carefully consider whether integration is necessary and feasible, and in cases where it is not, should explore the use of a *multi‐methods* approach instead (Anguera et al., 2018). A comprehensive review of the use of mixed methods in genetic counseling research has been recently conducted (Borle & Austin, 2025).

### 3.9 Telling stories with qualitative data

The beauty of qualitative research lies in its inherent creativity, and this extends to the reporting of the analysis. While there are several ways to construct narratives for qualitative data, many of the papers reviewed here reported their analyses as lengthy and fragmented results sections consisting of brief statements followed by supportive quotations. Rather than constructing a coherent narrative, in many cases, analyses were presented as itemized collections of codes, categories, subcategories, themes, or sub‐themes, thereby diluting the experiences they intended to illuminate (Sandelowski, 2007). This itemization gives the impression that the work is highly descriptive, the depth of analysis is shallow, and may reflect concerns regarding misrepresentation of findings, over analysis, or a lack of confidence in making interpretations (Antaki et al., 2003). Consideration of options (conceptual models; organizing diagrams; tables; poetry; arts‐based methods; metaphor analysis; oral histories; vignettes; infographics; and other visual representations) that could complement narrative prose would be beneficial in enhancing the depth of analysis beyond basic pattern recognition and category/theme development (Kara, 2022; Thorne, 2020). As discussed previously (see Section 3.1), the nature of a data gathering technique can inherently promote storytelling and multivocality. Data gathering techniques and the representation of findings should be considered thoughtfully and collectively to enhance these aspects.

### 3.10 Purpose: Describing, theorizing, or implementing

While not an explicit expectation outlined in the Journal's current author guidelines, the inclusion of clinical and practical implications was previously a requirement for authors. Indeed, this is a common practice in the field that aligns with the intentions of producing action‐oriented findings typical of an allied health discipline and may even be requested from authors by reviewers and editors. Among the papers reviewed, it is therefore not surprising that almost all authors provided this type of information even in the many cases in which findings were predominantly descriptive, rather than analytical, in nature. Moreover, theoretical framing (typically at the outset of the study or in the interpretation of results) was often absent or only superficially applied. This absence suggests a lack of methodological congruence in which the reported implications and intended use of the research (e.g., informing implementation or service delivery, or theorizing about practice) were misaligned. Descriptive studies can offer valuable insights, especially when exploring new or rare phenomena or the experiences of those with minoritized identities. However, when the intention is to make claims about implementation of findings, studies need to be designed and reported with a corresponding level of conceptual engagement and depth of interpretation. This has important implications for the ways in which qualitative analyses are perceived and used in applied health contexts (Thorne, 2020). Improving the reporting of these components (methodology in general, and the role of theory/theoretical frameworks) will enhance arguments for the validity of the practical implications being derived from qualitative work. The purpose of “describe,” “theorize,” or “implement” can also be previewed through well‐crafted research questions and aims.

## 4 RECOMMENDATIONS

### 4.1 Toward methodological congruence

Deliberate attention to the coherent alignment between research purpose, paradigmatic orientations, methodologies, reporting, and outputs is an essential first step in enhancing the quality of qualitative genetic counseling research. In Box 1 (part A), I have provided a list of 10 questions that can be used as an internal barometer of methodological congruence. The list begins with the explicit naming of the philosophical paradigm guiding the work and clearly articulating how the chosen paradigm supports the research questions and methodological choices. I suggest that the explicit naming and discussion of the underlying philosophy are equally important across small q, medium Q, and Big Q research (and across qualitative, quantitative, and mixed methods inquiries) in service to the goal of methodological congruence. Leaving information such as the paradigmatic orientation of the study or the specific type of mixed methods approach to be inferred or assumed by a reader can lead to misinterpretation of a study's underlying assumptions and rationale. This could in turn result in a weakening of the perceived coherence, credibility, or utility of the research. Precise rationales for decisions related to participant group selection, data gathering techniques, analysis, and reporting should follow.

BOX 1 **Questions to enhance the methodological congruence of qualitative genetic counseling research.**

- For the *conduct and reporting* of qualitative research:
  1. Have you stated clearly what philosophical paradigm you have used? If you have used more than one paradigm, have you provided sufficient justification to explain your decision including relevant citations?
  2. Have you explained how the philosophical assumptions of the chosen paradigm/s support the nature of your research question?
  3. Have you described the data gathering technique/s of your methodology clearly, including a justification for your choices?
  4. Have you described your data analysis method/s transparently, and thoroughly, including a justification/rationale for your choice/s and relevant citations?
  5. Have you explained and justified your participant group makeup using quality/rigor concepts that align with your paradigmatic choice?
  6. Have you described and implemented strategies to enhance the rigor of your study and presentation of your findings?
  7. Have you considered and implemented the best representation of your findings (e.g. graphically or in a table)?
  8. Have you discussed your findings in relation to theoretical knowledge in published literature?
  9. Have you discussed the broader implications of your findings to the extent that is appropriate for the paradigm you have chosen and the goal of your study?
  10. Have you included a discussion of reflexivity and positionality as it pertains to your background, identities, and perspectives and how they have influenced the research process and outcomes?
- For the *peer review* of qualitative research [based on (Clarke et al., 2025)]:
  1. Do you have sufficient expertise/familiarity to conduct a peer review of a qualitative research manuscript? [If the answer is no, consider declining the request to review or confine your review to the subject matter only and inform the editor of this decision].
  2. Have you evaluated the qualitative research within the context of the relevant qualitative values/philosophical paradigms/frameworks rather than applying quantitative and/or post‐positivist assumptions of quality and rigour?
  3. Have you evaluated the qualitative research approach used on its own merits rather than on the parameters of another/your preferred qualitative approach?
  4. Does the reporting of the qualitative method used align with a guideline specific to its use (e.g. RTARG) or to a generally applicable one (e.g. BQQRG)?

### 4.2 Toward knowingness

As previously described, knowingness refers to a researcher's ability to articulate what they are doing, why they are doing it, and how their choices make conceptual sense. It is not enough to populate methodological descriptions with terms and jargon that “sound right.” Researchers must understand and clearly communicate the meaning of those concepts within the chosen paradigm. If we consider that methods of scientific inquiry are like languages, then the terms used structure the ways of thinking and enacting. Adopting terminology and their associated processes, without truly understanding or being able to explain their meaning within a given system leads to superficial and often misleading accounts of the research process (Small, 2005). Researchers should avoid the temptation to legitimize qualitative work through the borrowed authority of quantitative language or by mimicking quantitative conventions. Rather, cultivating knowingness requires that researchers embrace qualitative inquiry as systems of thought that have their own languages and values. This shifts the focus to how researchers can do qualitative research well, rather than how they can make it appear more “scientific.”

Knowingness can be achieved through reflexive engagement, in which researchers critically reflect on philosophical assumptions, their positionalities, and their interactions with the data that shape the analysis (Braun & Clarke, 2019, 2021c). Specific activities that can be used to cultivate knowingness include keeping reflexive journals or memos that document decisions, assumptions, and evolving interpretations; engaging in critical dialogue with colleagues, mentors, or supervisors; explicitly articulating philosophical assumptions in the reporting of qualitative research to clarify how these inform analytic choices; and providing appropriate citations to support these philosophical and design decisions. All of this requires that researchers first learn and understand these concepts (see Section 4.4).

### 4.3 Toward transparency

Transparency is what enables a relationship to be formed between the audience and the findings of a qualitative research study. Engendering trust and integrity through transparency is achieved when researchers are open about their decisions and perspectives. Inviting readers into the meaning‐making processes allows them to better assess the relevance, resonance, and utility of the insights for their own contexts. Transparent reporting requires clear articulation of all methodological choices, including data generation techniques, analytical procedures, participant group makeup and rationales, with appropriate justifications and citations. This also includes disclosing and justifying departures from standard methodologies and offering positionality statements and evidence of reflexivity. Transparency should not be construed as replicability in the positivist sense, but rather openness about how meaning was co‐constructed and how conclusions were derived.

It is important to acknowledge that word count restrictions imposed by many journals often constrain the space available for the detailed methodology descriptions that are required to achieve methodological congruence and transparency. Over time, this can lead to researchers routinely condensing or omitting details, even when space is available. Remaining mindful of this tendency, and where appropriate, advocating for the inclusion of more space to provide fuller methodological reporting may be important strategies in navigating these challenges. Including a more fulsome description of methodology in an appendix or supplementary materials may be an appropriate interim solution.

The use of reporting guidelines can support transparency by offering structured prompts that help researchers ensure they have incorporated key components of their research process. These tools can be helpful in making the often‐intangible elements of qualitative research more overt. However, their indiscriminate use should be cautioned against as they tend to promote a checklist mentality that may limit deep engagement with underlying concepts (Braun & Clarke, 2024b). Furthermore, some guidelines are better suited to particular paradigms (e.g., COREQ aligns with a positivist paradigm while BQQRG aligns with Big Q research). Applying guidelines without due thought to their alignment with paradigms and data analysis approaches is a risk to methodological congruence.

### 4.4 The teaching of qualitative research methods

The contribution of genetic counseling students to the research being conducted within our community is immense (Zayhowski et al., 2024). Given the propensity for qualitative genetic counseling research to be conducted by trainees, there is a clear need for enhanced training in qualitative methodologies. Many of the reviewed studies exhibited limited methodological diversity and a reliance on basic descriptive techniques, often chosen for their feasibility rather than their conceptual fit. I am a strong advocate for doing what is pragmatic, and as a supervisor of genetic counseling student research, I am aware of the constraints we need to place on research to ensure its feasibility in that context. However, I also believe that we need to make decisions about qualitative research design by thoughtfully considering what offers us the best possible answers to the questions that drive inquiry in the first place, rather than what is expediently achieved. This endeavor must go hand in hand with a need to bolster student education and training in qualitative research.

One particularly challenging obstacle is that like in most health professions, genetic counseling trainees are typically introduced to research through almost exclusively quantitative, positivist, or scientific realist frameworks that emphasize objectivity, standardization, and generalizability as markers of quality (Piemonte, 2018). Entering the Big Q research environment, with its embrace of subjectivity, interpretation, and partiality, thus requires a profound epistemological and conceptual shift. Helping trainees make this shift involves not only teaching qualitative methods as a set of techniques but also engaging with the philosophical and values‐based foundations that underpin qualitative inquiry. In addition to teaching about interpretivist, constructivist, pragmatic, critical, cultural, postmodern, post‐structural, and other paradigms, exposure to exemplars of high‐quality qualitative research and structured reflexivity exercises, mentorship that supports grappling with the discomfort of these epistemological and ontological transitions would be imperative. This would better prepare emerging researchers to design coherent studies that move qualitative research beyond surface‐level description toward highly impactful, theoretically informed, rigorous genetic counseling analyses.

### 4.5 Strengthening editorial and peer review practices

While much of this review has focused on the reporting of qualitative research from the perspective of the researchers themselves, it is essential to acknowledge that the publication of high‐quality research does not only lie in their hands, but also those of reviewers and editors. Many incongruences reported here may have either been overlooked or inadvertently encouraged by reviewers and editors unfamiliar with Big Q approaches. The concept of methodological congruence remains an important principle in ensuring quality from this perspective. Box 1 (part B) outlines considerations for the review of qualitative research based on the findings of this review as well as (Clarke et al., 2025). These considerations include an imperative to be introspective about the limitations of one's qualitative research knowledge, understand paradigmatic distinctions, recognize appropriate quality practices, and avoid imposing quantitative expectations of quality onto qualitative inquiries.

## 5 CONCLUSION

Qualitative research has become an essential component of genetic counseling scholarship. Yet, its potential is limited when we fail to ensure the highest quality standards in conducting, reporting, and reviewing these works. This review underscores the need for a cultural and structural shift in how qualitative research is taught, conducted, enacted, and published in the field of genetic counseling. It is also important to acknowledge the inherently interpretive, intuitive, and artistic nature of qualitative research and not be tempted to reduce this form of inquiry to a set of technical procedures. Imposing rigid templates on the reporting of qualitative research risks losing its nuance and richness. By honoring both creativity and clarity of reporting, we can elevate the standing of qualitative research in applied health contexts and ensure its enhanced relevance in genetic counseling practice.

## AUTHOR CONTRIBUTIONS

**Tasha Wainstein:** Conceptualization; formal analysis; methodology; project administration; writing – original draft; writing – review and editing.

## CONFLICT OF INTEREST STATEMENT

Tasha Wainstein is the Deputy Editor for the *Journal of Genetic Counseling* and the author of this article. She was excluded from editorial decision‐making related to the acceptance of this article for publication in the *Journal*.

## ETHICS STATEMENT

Animal studies: This manuscript did not include data from animal studies.

Human studies and informed consent: No patients or participants participated in this work, and informed consent was not required.

## AI STATEMENT

Artificial Intelligence Generated Content (AIGC) tools were not used in the development of any portion of this manuscript.

## Supporting information


Appendix S1

## Data Availability

No data were generated for the purposes of this article.

## Footnotes

^1^
Quality and rigor are not mutually exclusive concepts within qualitative inquiry. Generally, the term “rigor” refers to the conduct of research and “quality” to the reporting of that research (Braun & Clarke, 2024b), but they are frequently used interchangeably. While quality and rigor in quantitative inquiries are almost uniformly considered to be assessed through validity, reliability, objectivity, and generalizability, defining and assessing quality in qualitative research needs to be discussed in ways that are particular to the paradigm or even specific data analysis approach used. For a comprehensive discussion see (Flick, 2018).

^2^
“Methodological congruence” (Braun & Clarke, 2024c) has also been described as, or has overlapping understanding with concepts such as, “methodological coherence” (Braun & Clarke, 2023a), “methodological integrity” (Levitt et al., 2017), “meaningful coherence” (Tracy, 2010), and “paradigmatic integrity” (Hills, 2000).

^3^
“Transparency” (Tuval‐Mashiach, 2017) is an overlapping concept with “sincerity” (Tracy, 2010), “authenticity” (Guba & Lincoln, 2005), and “reflexive openness” (Braun & Clarke, 2024b).

^4^
The term “Analysis” has been intentionally used here to reflect the interpretive nature of qualitative research. In the case of this review, I generated this analysis subjectively, based on analytical engagement, reflexivity, and attention to the literature. Using the term “analysis” over “results” or “findings” therefore helps distance the work from connotations of objectivity or discovery‐oriented intentions typical of quantitative or post‐positivist paradigms (Braun & Clarke, 2024b).

^5^
Some researchers have conceptualized phenomenology and grounded theory as philosophical paradigms for qualitative research. However, since they are more typically described and operationalized as methodologies, it is important that those wishing to conceptualize their study's worldview in this manner, provide coherent and adequate rationalizations for these decisions (in relation to epistemology, ontology, and axiology), including relevant citations.

^6^
Use of the terms “participant group” and “recruitment” or “selection” over “sample” and “sampling strategies” reflects my alignment with non‐positivist paradigms of qualitative research that seek to differentiate these activities from positivist notions of statistical generalization (Braun & Clarke, 2024a).

^7^
Specific concerns with the use of saturation as a justification for participant group size and sufficiency have been reported extensively (Braun & Clarke, 2021d; Vasileiou et al., 2018; Wainstein et al., 2023).

^8^
There are, of course, exceptions: it is possible that lengthy, rich, and detailed responses are produced in response to open‐ended questions that can be analyzed independently of a quantitative survey. See for example (Hendry et al., 2025) in which inductive content analysis was used to analyze over 28,000 words from nearly 400 survey participants in response to two open‐ended questions. Also, surveys can use exclusively or predominantly open‐ended questions, and when designed intentionally, are compatible with using Big Q approaches like RTA (Braun et al., 2021).
