# Supplementary material for: Qualitative research reporting in genetic counseling: A state‐of‐the‐art assessment and recommendations for enhancing methodological congruence and quality
Source: J Genet Couns. 2026 Feb 15;35(1):e70160. doi: 10.1002/jgc4.70160 (PMC12907537; doi:10.1002/jgc4.70160)
Supplement: Supplementary file 1 — Appendix S1 [file JGC4-35-0-s001.docx]

# Supplementary Materials

## Qualitative research reporting in genetic counseling: a state-of-the-art assessment and recommendations for enhancing methodological congruence and quality

Tasha Wainstein, PhD, MSc (Med), CCGC | [tasha.wainstein@bcchr.ca](mailto:tasha.wainstein@bcchr.ca)

### Glossary

- **Analytical notes (also memos; field notes):** Written reflections made by researchers at any point during the research process (including data gathering, and analysis stages) which capture insights, ideas, emerging patterns, and reflexivity. These can be used in helping to shape the final analysis and making the research process more transparent.
- **Axiology**: Branch of philosophy that studies values and ethics in research. Engaging with axiology requires researchers to think about their own values and ethical stances that are (or should be) guiding the research, and what values or outcomes should result from their research. For example, axiological questions might include, “can research be value neutral?” or “are we just seeking to understand something or should the goal of the research be to create some kind of change?”
- **Categories (as used in content analysis)**: Broad groupings of similar codes or meanings derived from qualitative data. Categories are used to organize data into meaningful clusters for interpretation.
- **Collaborative coding (or co-coding):** A process in which two or more researchers code the same dataset to achieve an enhanced understanding of the underlying meaning of the data from multiple perspectives. A quality practice that is aligned with constructivist / “Big Q” approaches. See: Coulston F, Lynch F, Vears DF (2025). Collaborative coding in inductive content analysis: Why, when, and how to do it. *Journal of Genetic Counseling,* 34(3):e70030, <https://doi.org/10.1002/jgc4.70030>
- **Consensual qualitative research (CQR) framework**: An approach to qualitative research that combines positivist and constructivist paradigms and emphasizes multiple researchers working collaboratively to reach consensus on coding and interpretation. See: Hill CE and Knox S (2021). Essentials of Consensual Qualitative Research. American Psychological Association, <https://doi.org.10.1037/0000215-001>.
- **Consensus coding**: A process in which two or more researchers independently apply codes to data segments and then discuss their coding to achieve agreement or consensus on the appropriateness and accuracy of the codes. A quality practice used commonly in positivist paradigms to enhance trustworthiness and objectivity.
- **“Content analysis”**: A group of analysis procedures used for analyzing text-based data based on observable patterns in the manifest (apparent; descriptive) and/or latent (inferential; interpretive) content. Content analysis can be quantitative or qualitative; qualitative content analysis can be further categorized as either deductive or inductive. It is important to provide a clear description of the specific type of content analysis to ensure methodological congruence. See: Lynch F, Gillam L, Vears DF (2023). Alleviating the confusion around content analysis: a comment in response to Wainstein, Elliott, & Austin 2023. *Journal of Genetic Counseling,* 33:1126-1129, <https://doi.org/10.1002/jgc4.1829>.
- **Crystallization**: A framework for conducting research that combines multiple forms of analysis across the spectrum of philosophical paradigms and multiple representations of data (scientific and artistic) to produce complex and nuanced accounts of a phenomenon. A quality practice associated with Big Q approaches. See: Ellingson LL (2014). “The truth must dazzle gradually”: enriching relationship research using a crystallization framework. *Journal of Personal and Social Relationships,* 31(4):442-450. <https://doi.org/10.1177/0265407514523553>.
- **Epistemology**: Branch of philosophy that studies knowledge and how it is acquired. Epistemological views span from the idea that knowledge exists independently of us and is waiting to be discovered, to knowledge is constructed by researchers and their research participants based on experiences and perceptions.
- **Grounded theory**: A group of data analysis procedures that share the goal of investigating phenomena for which there is little to no existing theory such that the main outcome of a study is a substantive theory grounded in the data. Types of grounded theory differ according to their underlying philosophical leanings with post-positivist and constructivist types being the most common. See: Fishler Malone K, Carmichael N (2025). Research methodologies in genetic counseling: grounded theory. *Journal of Genetic Counseling,* 34(3):e70060. <https://doi.org/10.1002/jgc4.70060>.
- **Information power**: A tool that can be used to assess the appropriateness of dataset size and scope with respect to the research question. Information power considers (i) study aim, (ii) sample specificity, (iii) use of established theory, (iv) quality of dialogue, and (v) analysis strategy to inform a subjective, interpretive, and partial view of the research’s capacity to respond to its aims. See: Malterud K, Siersma VD, Guassora A (2016). Sample size in qualitative interview studies: guided by information power. *Qualitative Health Research,* 26(12):1753-1760. <https://doi.org/10.1177/1049732315617444>.
- **Intercoder reliability**: A “small q” quality practice approach in which the degree to which researchers agree on how to code the same qualitative dataset is measured. It is frequently used to test the consistency and validity of a codebook through statistical measurements (e.g. Holsti’s method or Cohen’s kappa).
- **Interpretative phenomenological analysis (IPA)**: An approach to data analysis in which an understanding of the research participant’s subjective, first-hand knowledge and experiences is foregrounded. See: Starr RA, Smith JA (2025). Interpretative phenomenological analysis and genetic counseling. *Journal of Genetic Counseling,* 34(3):e70061. <https://doi.org/10.1002/jgc4.70061>.
- **Interpretive description**: A pragmatically oriented qualitative methodology which intends to generate practical understanding of complex clinical phenomena. It is rooted in applied health research and emphasizes contextual relevance and practice-based insights. See: Thorne S (2016). Interpretive description: Qualitative research for Applied Practice. *Taylor & Francis.*
- **Knowingness**: A researcher’s ability to articulate what they are doing, why they are doing it, and how their choices make conceptual sense. Braun V, & Clarke V(2024). A critical review of the reporting of reflexive thematic analysis in Health Promotion International. *Health Promotion International*, *39*(3). <https://doi.org/10.1093/heapro/daae049>.
- **Member checking**: A “small q” quality practice in which data or interpretations thereof are returned to research participants to elicit their perspectives on the data as a means of correcting errors, eliminating misinterpretations, and ensuring its accuracy and credibility. See: Varpio L, Ajjawi R, Monrouxe LV, et al (2017). Shedding the cobra effect: problematising thematic emergence, triangulation, saturation, and member checking. *Medical Education,* 51:40-50. <https://doi.org/10.1111/medu.13124>.
- **Member reflections:** An iterative, dialogic process in which research participants are invited to reflect, expand, or complicate the researcher’s developing interpretations. The intention is to create additional opportunities for co-construction of meaning between participants and researchers to enhance interpretive richness in “Big Q” paradigms. See: Tracy SJ. (2010). Qualitative Quality: Eight “big-tent” criteria for excellent qualitative research. *Qual Inquiry*, 16:837-851. <https://doi.org/10.1177/1077800410383121>.
- **Method**: The specific techniques used to gather (e.g. interviews, surveys, focus groups, document analysis) and analyze (e.g. inductive content analysis, reflexive thematic analysis; narrative inquiry) data. The term “method” is not interchangeable with the term “methodology” but rather form part of your methodology. It is also important to note that certain methods may encode particular philosophical assumptions that need to be considered in study design. For example, coding reliability thematic analysis uses techniques that align with a positivist (small q) paradigm, while reflexive thematic analysis aligns with a constructivist (Big Q) paradigm.
- **Methodological congruence**: A study is methodologically congruent when its purpose, research questions, assumptions, data gathering and analysis procedures, and outputs are all in alignment with another when oriented around philosophical paradigms. “Methodological congruence” (Braun and Clarke, 2024) has also been described as, or has overlapping understanding with concepts such as, “methodological coherence” (Braun and Clarke 2023), “methodological integrity” Levitt et al, 2017), “meaningful coherence” (Tracy, 2010), and “paradigmatic integrity” (Hills, 2000).
- **Methodology**: The strategic and philosophical aspects of research design which guide the approach to the study. Methodologies specify the guiding theoretical assumptions, suitable research questions, and ideal methods of data gathering and analytical procedures. The methodology should also demonstrate congruence between the design of the inquiry and the desired outcomes.
- **Mixed methods research**: An approach to research that incorporates gathering both qualitative and quantitative data and crucially, the integration of results from these two types of data. Mixed methods research is useful in situations where the use of either type of approach is inadequate to achieve the goals of inquiry. See: Borle K, Austin J (2025). Using mixed methods for genetic counseling research. *Journal of Genetic Counseling,* 34(3):e70031. <https://doi.org/10.1002/jgc4.70031>.
- **Multi-method research**: The use of more than one method (qualitative or quantitative) in a single study without integrating the findings. See: Anguera MT, Blanco-Villasenor A, Losada JL, et al (2018). Revisiting the difference between mixed methods and multimethods: it is all in the name? *Quality & Quantity,* 52:2757-2770. <https://doi.org/10.1007/s11135-018-0700-2>.
- **Ontology**: Branch of philosophy that studies the nature of reality and what can be known of reality. Ontological views may vary from there being a single fixed reality that exists externally to the observer, to there being multiple realities which are subjectively constructed by each observer.
- **Philosophical paradigm**: A framework of beliefs about knowledge (epistemology), reality (ontology), axiology (values), and methodology (design) that guides the research process including data gathering, data analysis approaches, and the construction of the outputs of the analysis. The most common examples include (post)positivism, constructivism/interpretivism, critical theory, and pragmatism. Explicitly stating the philosophical paradigm in which a study has been conducted is an integral first step to achieving methodological congruence in the reporting of qualitative research studies. See: Wainstein T, Elliott AM, Austin J (2023). Considerations for the use of qualitative methodologies in genetic counseling research. *Journal of Genetic Counseling,* 32(2):300-314. <https://doi.org/10.1002/jgc4.1644>.
- **Positionality**: The embodied relationship between the researcher’s socially constructed identity and the existing systems of oppression and/or privilege in which they conduct their work. Recognition that a researcher’s personal and professional experiences, social identities, values, and biases is important across all philosophical paradigms of research. The way in which positionality is enacted though differs; in positivist paradigms, positionalities are considered in so far as they need to be mitigated to enhance objectivity of the work. While in non-positivist paradigms, researchers’ positionalities are viewed as a resource to enhance the quality of the research. See: Zayhowski K, Borle K, Channaoui N (2025). Enhancing rigor and justice in genetic counseling research with reflexivity and positionality. *Journal of Genetic Counseling,* 34(3):e70066. <https://doi.org/10.1002/jgc4.70066>.
- **Quality practices**: A suite of practices that are used in qualitative research to ensure and enhance the quality and rigor of the work being conducted. The choice of which practice/s to use is dependent upon the paradigm within which the research is being conducted. Quality practices should be articulated clearly in the methodology section of a qualitative research report.
- **Reflexivity**: The process through which researchers become aware of their positionalities and the ways these play out in their understanding and analysis of the research. Engaging with reflexivity throughout the research lifecycle is an important means of ensuring that power imbalances inherent to the researcher-participant interaction are mitigated. See: Zayhowski K, Borle K, Channaoui N (2025). Enhancing rigor and justice in genetic counseling research with reflexivity and positionality. *Journal of Genetic Counseling,* 34(3):e70066. <https://doi.org/10.1002/jgc4.70066>.
- **Saturation**: A “small q” quality practice originally developed in grounded theory to indicate the participant group is of sufficient size, the dataset is complete, or the objective truth has been discovered. Saturation describes the state in which no new or additional information, codes, or ideas can be elicited from the data.
- **(Statistical-probabilistic) generalizability**: While there are many types, use of the term “generalizability” on its own either knowingly or unintentionally refers to the idea that a study’s results can be extrapolated to a wider population because of the use of statistical sampling procedures. This is a corner stone of quality in quantitative research, but other types of generalizability (naturalistic, inferential, analytic, intersectional) are important for consideration in qualitative research. See: Smith B (2018). Generalizability in qualitative research: misunderstandings, opportunities and recommendations for the sport and exercise sciences. *Qualitative Research in Sport, Exercise and Health*, *10*(1), 137–149. <https://doi.org/10.1080/2159676X.2017.1393221>.
- **“Thematic analysis” (TA):** A group of data analysis approaches which share some similarities with respect to the development of patterns across cases but also have some differences with respect to their underlying philosophical paradigms. The most commonly known approaches include coding reliability TA, codebook TA, and reflexive TA. Reporting the specific type of TA that was used and demonstrating alignment with the appropriate philosophical paradigm is essential to achieve methodological congruence. See: Braun V, Clarke V (2021). Can I use TA? Should I use TA? Should I *not* use TA? Comparing reflexive thematic analysis and other pattern-based qualitative approaches. *Counseling and Psychotherapy in Research,* 21(1):37-47. <https://doi.org/10.1002/capr.12360>.
- **Theme**: While the definition of a theme may differ according to distinctive analysis approaches, in general it refers to a way of capturing the patterns which have been developed from the dataset, usually with relevance to answering the research question. In reflexive thematic analysis for example, themes capture shared meaning underpinned by a central concept. In other types of TA though, themes can resemble categories or topic summaries in which all data relevant to a topic are grouped together and may not represent a story created from the data.
- **Theoretical sufficiency**: A way of defining the end point of data collection or analysis by establishing the point at which there is sufficient dept of understanding to allow for the development of a theory. The implication is that beyond this point, it is still possible to achieve new insights and that the presented information is, by design, a subjective and partial analysis. See: Dey I (1999). Grounding grounded theory: guidelines for qualitative inquiry. *Academic Press.*
- **Thick description:** A detailed, context-rich account of setting, experiences, or interactions beyond surface-level reporting. The aim of providing a thick description is to help convey the context of constructed meanings of data so that readers / knowledge users can make decisions about the transferability of the analysis.
- **Transferability (or inferential generalizability)**: A process that occurs when knowledge users in one setting consider taking up the implications of a research study by considering the extent to which the findings are transferable to other settings. Researchers should aim to provide rich description and accessible writing to allow knowledge users to consider transferability. Smith B (2018). Generalizability in qualitative research: misunderstandings, opportunities and recommendations for the sport and exercise sciences. *Qualitative Research in Sport, Exercise and Health*, *10*(1), 137–149. <https://doi.org/10.1080/2159676X.2017.1393221>.
- **Transparency**: The researcher/s responsibility to clearly and openly articulate the research procedures and findings such that the knowledge user does not have to make assumptions about the quality/rigor of the work. See: Tuval-Mashiach R (2017). Raising the curtain: The importance of transparency in qualitative research. *Qualitative Psychology*, *4*(2), 126–138. <https://doi.org/10.1037/qup0000062>.
- **Triangulation**: A “small q” quality practice in which researchers gather multiple sources of information pertaining to the research question as a way of converging on a singular, objective truth. Triangulation strategies include data, investigator, theory, and method triangulation. See: Morgan H (2024). Using triangulation and crystallization to make qualitative studies trustworthy and rigorous. *The Qualitative Report,* 29(7):1844-1856. <https://doi.org/10.46743/2160-3715-2024.6071>.

**Table S1** Paper characteristics for the 34 studies published in the Journal of Genetic Counseling in 2023 (Volume 32; Issues 1-6) included in the critical review of qualitative methodologies

| **Paper Characteristic** | **N** |
| --- | --- |
| *Study Design* | |
| Mixed methods | 6 |
| Open-ended questions in a quantitative survey | 7 |
| Qualitative | 21 |
| *Data Collection Technique*^1^ | |
| Focus group | 2 |
| Interview | 24 |
| Multiple | 6 |
| Other (live chat transcript) | 1 |
| Survey (open-ended questions; story completion prompt) | 13 |
| *Data Analysis Method*^1^ | |
| Content analysis | 8 |
| Consensual Qualitative Research Framework | 1 |
| Grounded theory | 2 |
| Interpretive description | 3 |
| Not named (or vague description) | 11 |
| Thematic analysis | 9 |
| *Participant Group*^1^ | |
| Genetic counseling students | 5 |
| Genetic counselors^2^ | 16 |
| Multiple | 8 |
| Other healthcare professionals^3^ | 7 |
| Patients/clients who sought genetic counseling services^4^ | 13 |
| *Research Question Focus* | |
| Behaviours | 3 |
| Lived experiences | 5 |
| Needs and concerns | 3 |
| Opinions | 1 |
| Perspectives | 15 |
| Professional experiences | 7 |
| *Genetic Counseling Trainee is First Author* | |
| No | 8 |
| Yes | 26 |
| *Participant Group Size Range* | |
| All | 9-258 |
| Exclusively qualitative studies | 9-35 |
| Qualitative studies + qualitative portions of mixed methods | 9-136 |
| *Median Participant Group Size (IQR)* | |
| All | 24.5 (16-106.5) |
| Exclusively qualitative studies | 20 (13-25) |
| Qualitative studies + qualitative portions of mixed methods | 20 (13.5-25) |

1. Totals equal greater than 34 as several studies used more than one data collection technique/analysis method or included more than one participant group.
2. Includes specific groups of genetic counselors (e.g. supervisors, training program leadership).
3. Clinical and laboratory geneticists, genetics residents, occupational therapists, interpreters, volunteers, patient co-ordinators, nurses, hospital leadership.
4. Cancer, prenatal, carriers, DTC-GT, Disability community, LGBTQ+ community, parents, siblings.
